# Cardiovascular disease, associated risk factors, and risk of dementia: An umbrella review of meta-analyses

**DOI:** 10.3389/fepid.2023.1095236

**Published:** 2023-02-09

**Authors:** Jacob Brain, Leanne Greene, Eugene Y. H. Tang, Jennie Louise, Amy Salter, Sarah Beach, Deborah Turnbull, Mario Siervo, Blossom C. M. Stephan, Phillip J. Tully

**Affiliations:** ^1^ Institute of Mental Health, School of Medicine, University of Nottingham, Innovation Park, Jubilee Campus, Nottingham, United Kingdom; ^2^ Freemasons Foundation Centre for Men’s Health, Discipline of Medicine, School of Psychology, The University of Adelaide, Adelaide, SA, Australia; ^3^ Population Health Sciences Institute, Faculty of Medical Sciences, Newcastle University, Newcastle upon Tyne, United Kingdom; ^4^ Discipline of Obstetrics & Gynaecology, the Robinson Research Institute, The University of Adelaide, Adelaide, SA, Australia; ^5^ School of Public Health, Faculty of Health and Medical Sciences, University of Adelaide, Adelaide, SA, Australia; ^6^ University of Nottingham Libraries, University of Nottingham, King’s Meadow Campus, Nottingham, United Kingdom; ^7^ School of Life Sciences, The University of Nottingham Medical School, Queen's Medical Centre, Nottingham, United Kingdom; ^8^ Faculty of Medicine and Health, School of Psychology, University of New England, Armidale, NSW, Australia

**Keywords:** dementia risk, cardiovascular disease, umbrella review, epidemiology, protective factor, dementia, risk factors

## Abstract

**Introduction:**

Cardiovascular diseases (CVDs) have been associated with an increased risk of dementia; yet the evidence is mixed. This review critically appraises and synthesises current evidence exploring associations between dementia risk and CVD and their risk factors, including coronary heart disease, heart failure, atrial fibrillation, hypertension, hyperlipidaemia, and arterial stiffness.

**Methods:**

MEDLINE, Embase, PsycINFO, and the Cochrane Database of Systematic Reviews were searched to identify systematic reviews with meta-analyses investigating the association between at least one of the CVDs of interest and dementia risk. The Joanna Briggs Institute (JBI) Critical Appraisal Checklist for Systematic Reviews was used to assess methodological quality.

**Results:**

Twenty-five meta-analyses published between 2007 and 2021 were included. Studies largely consisted of cohorts from North America and Europe. Findings were variable, with coronary heart disease, heart failure, and atrial fibrillation consistently associated with increased risk for all-cause dementia, but results were inconsistent for Alzheimer's disease. Hypertension was more frequently associated with dementia during mid-life compared to late life. Findings concerning cholesterol were complex, and while results were inconsistent for low-density lipoprotein cholesterol and total cholesterol, there appeared to be no associations between triglycerides and high-density lipoprotein cholesterol. All meta-analyses investigating hypercholesterolaemia showed significant increases in dementia risk. There was a paucity of research on the association between arterial stiffness and dementia risk.

**Conclusion:**

Targeted CVD dementia prevention strategies could reduce dementia prevalence. Future research should determine the underpinning mechanisms linking heart and brain health to determine the most effective strategies for dementia risk reduction in CVD populations.

## Introduction

Dementia is a global public health priority with approximately 55 million individuals living with the condition, with this number expected to rise to 139 million by 2050 (1, 2). These projected statistics reflect increasing human life expectancy rates (3), and the heightened risk of those in the oldest age brackets developing dementia (4). Increased longevity is largely related to the improvement in healthcare systems, yet there is debate as to whether individuals are spending their longer lifespans in better or worse health (5). Dementia has significant healthcare, economic and societal costs (6, 7). Therefore, in the absence of a cure, there is a need to identify at-risk populations to inform the development of prevention and risk reduction strategies.

There is evidence that dementia, Alzheimer's disease (AD) and vascular dementia (VaD) are associated with cardiovascular diseases (CVDs) and their risk factors, including coronary heart disease (CHD), heart failure (HF), atrial fibrillation (AF), hypertension, hyperlipidaemia, and arterial stiffness (8–10). The Lancet commission reported that hypertension and its risk factors in midlife (obesity and physical inactivity) account for approximately 2%–9.6% of dementia cases globally (11). However, overall findings have been inconsistent regarding CVD and dementia risk, especially in the case of hypertension at different stages in the life-course (12) and hyperlipidaemia (13). Indeed, it is unclear which CVDs consistently increase dementia risk and the potentially modifying role age may have. Therefore, the objective of this review was to synthesise the available evidence regarding the associations between CVDs and dementia.

## Methods

The review protocol was registered on PROSPERO (International Prospective Register of Systematic Reviews, Reference number: CRD42021265363) and has also been published (14). The review was completed in line with the Preferred Reporting Items for Systematic Reviews and Meta-Analyses (PRISMA) Guidelines (15). An information specialist (SB) supported the electronic literature search.

### Search strategy

MEDLINE, Embase, PsycINFO and the Cochrane Database for Systematic Reviews were searched from inception to the 10th October 2021. MeSh terms (e.g., “Dementia”, “Heart Failure”, “Hypertension”, “Arrhythmias”, “Hypercholesterolaemia”, “Lipids”, and “Vascular Stiffness”), keywords, and subject headings were used together with Boolean operators of “OR” and “AND”. Search filters were employed to further the retrieval of systematic reviews/meta-analyses (16). Backwards citation chaining was also utilised after full-text retrieval to ensure all relevant studies were captured. Searches were tailored to each database (Supplementary Table S1).

### Eligibility criteria

Eligible studies included reviews with meta-analyses investigating the association between incident dementia (all-cause and its subtypes, including for example AD and VaD) and one or more of the following six conditions in humans, including: coronary heart disease (CHD), heart failure (HF), atrial fibrillation (AF), hypertension, hyperlipidaemia, or arterial stiffness. As hyperlipidaemia is a broad disease category, we decided to include measures that focused on overall hypercholesterolemia, serum levels of total cholesterol (TC), low-density lipoprotein cholesterol (LDL-C), high-density lipoprotein cholesterol (HDL-C), and triglycerides (TG). Reviews that focused on mild cognitive impairment (MCI), stroke, or other neurological conditions at baseline were included if they performed separate analyses where dementia was the sole dependent variable. The systematic reviews/meta-analyses had to include either longitudinal cohort (i.e., prospective or retrospective) or case-control (e.g., nested case-control) study designs. Reviews focused specifically on clinical studies including trials and intervention studies were excluded. Editorial, narrative reviews, opinion pieces, and reviews of cross-sectional studies were also excluded. Articles had to be published in English. Reviews were considered for inclusion if they fulfilled the following criteria:

*Population*: The general population with or without a history of CVD or its associated risk factors, with information on incident dementia status. In line with similar reviews (17), we included all ages at baseline. Indeed, the strongest associations with dementia risk are found in those with poor cardiovascular health in midlife, with often mixed results in later-life cohorts (18, 19). Individuals were excluded if they received a diagnosis of dementia before the age of 65 (e.g., individuals with young- or early-onset dementia) due to this particular clinical group having potentially different aetiologies and disease trajectories (20).
*Exposure*: A diagnosis of any of the following conditions: CHD, HF, AF, hypertension, hyperlipidaemia, or arterial stiffness. These conditions were either self-reported or clinically diagnosed.
*Outcome*: All-cause dementia and its subtypes, including AD and VaD, diagnosed through operationalised diagnostic criteria in accordance with established criteria such as the Diagnostic and Statistical Manual of Mental Disorders (21), International Classification of Diseases (22), or a consensus diagnosis panel (23).

### Review selection

All references were imported into Covidence and duplicates removed using the automatic de-duplication function (24). Two researchers (JB and ET) independently screened title/abstracts against the eligibility criteria. All eligible references from the initial screening were reviewed in full to determine inclusion, independently by the same two researchers (JB and ET). Backwards citation searching of the references lists of included studies was also undertaken to identify potentially missed reviews (JB). Conflicts regarding inclusion were resolved by a third independent reviewer (BS).

### Data extraction

The Joanna Briggs Institute (JBI) data extraction form (25) was adapted to capture relevant study details including lead author and year of publication; cardiovascular disease of interest and definition of condition; dementia and diagnostic criteria; population characteristics and numbers; search and source details; date range of included studies; number and type of primary studies included; critical appraisal tools used; primary study countries; and results of meta-analyses. Corresponding authors were contacted by e-mail if full-text articles were not available, or information was missing. Data extraction was performed by two independent reviewers (JB and LG), with discrepancies resolved through discussion.

### Quality appraisal

The methodological quality of included studies was assessed independently by two reviewers (JB and LG) using the JBI Critical Appraisal Checklist for Systematic Reviews and Research Syntheses (26). This checklist has 11 items that cover potential biases in the review's design, conduct, and analysis which are scored as “Yes”, “No”, “Unclear”, or “N/A”. Items covered aspects of appropriate search strategies, the use of critical appraisal, data extraction methods, publication bias, and future directions in practice and policy. Disagreement between reviewers was resolved by discussion.

### Data synthesis

Due to the large scope of the review and substantial heterogeneity (e.g., different study designs and types of effect estimates and use of unadjusted and adjusted effect estimates) in included studies, a meta-meta-analysis was not possible. Therefore, a narrative synthesis was performed, with results presented in tabular and visual formats. For each of the CVD and risk factor investigated, results were presented separately. This umbrella review focuses on meta-analytical findings due to its regarded level of evidence.

## Results

**Figure 1:**
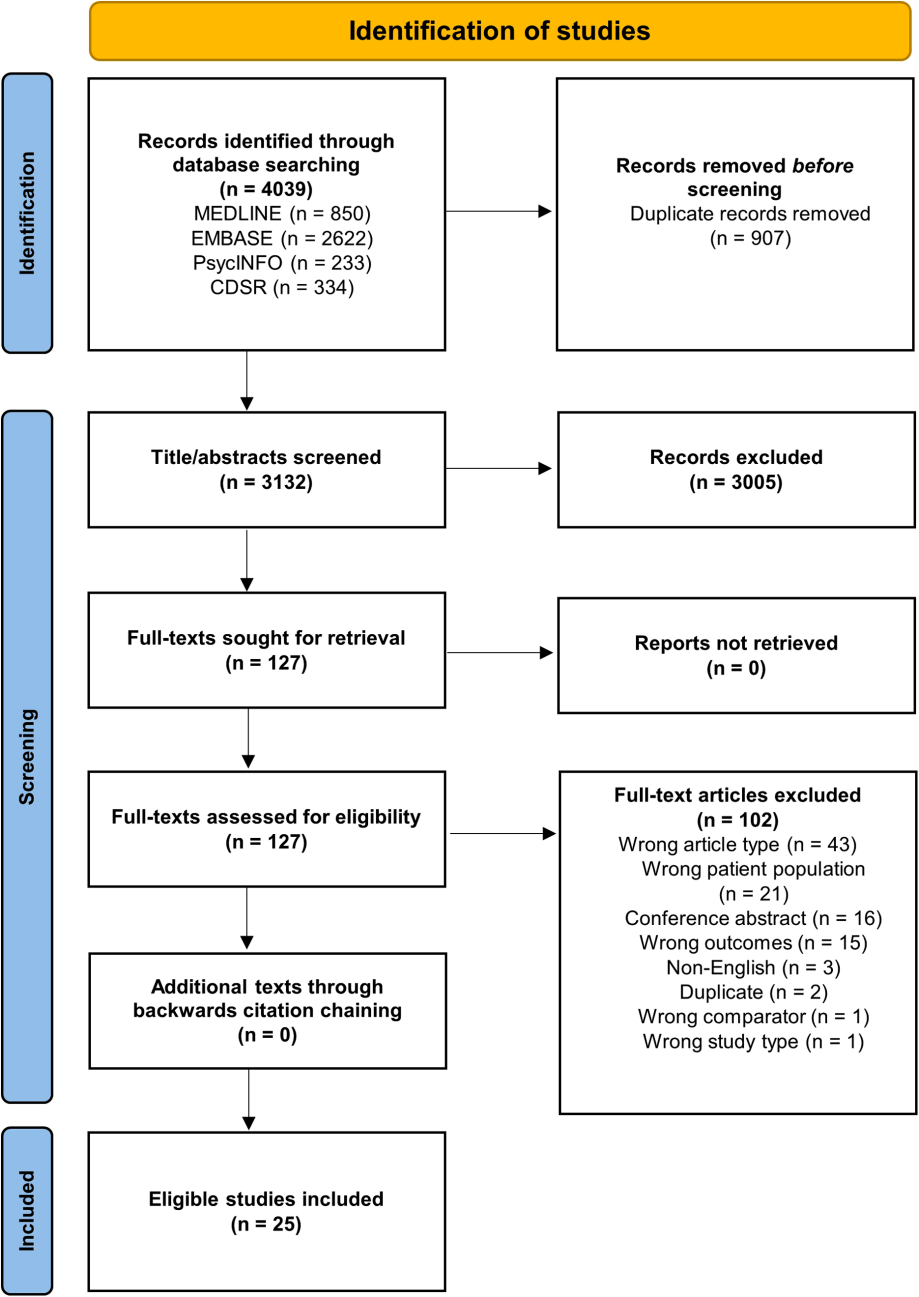
Study selection flowchart.

Of the 4,039 records identified from the electronic search, 25 meta-analyses (published from 2008 to 2021) met the inclusion criteria (Figure 1). The number of primary studies in each meta-analysis ranged from six to 351 that were published between 1985 and 2021, with the overall number of participants included in meta-analyses ranging from 3,335 to >3 million. Most studies were from sites in high-income countries (HICs) with few low- and middle-income countries (LMICs) represented, with the exception of Nigeria, Brazil, Iran, India, China, Turkey, Jordan, and Serbia (Table 1; Supplementary Table S2).

**Table 1:** Overview of meta-analyses.

Author and year	CVD condition	Outcome measure	Number of included studies	Included primary study designs	Countries of included primary studies	Key findings
Anstey (2008)	HL	Any	18	Prospective cohort	NR	High midlife TC consistently associated with dementia
Anstey (2017)	HL	Any dementia	17	Prospective cohort	USA, Sweden, France, Japan, the Netherlands, Australia, Finland, Italy	High midlife TC increased risk of late-life dementia, with no associations found in late-life. HDL measured in mid- and late-life was not associated with dementia
Deckers (2017)	CHD	All-cause	24	Prospective cohort, cross-sectional, and case-control	NR	CHD prospectively associated with an increased risk of dementia
Guan (2011)	HBP	AD	12	Longitudinal cohort and RCTs	USA, Sweden, Canada	Both hypertension and antihypertensive medication use not associated with the increased risk of AD
Islam (2019)	AF	All-cause AD	16	Longitudinal cohort, case-control and RCTs	Finland, USA, Italy, Sweden, UK, Taiwan, Netherlands	AF associated with an increased risk of all-cause dementia and AD
Kwok (2011)	AF	All-cause	15	Prospective cohort (and one RCT)	Italy, Spain, USA, UK, Finland, Sweden, Israel	AF evidenced to increases the risk of all-cause dementia
Lennon (2019)	HBP	AD	7	Prospective cohort and nested case-control	Norway, USA, Taiwan, Finland, South Korea, Japan	Midlife systolic hypertension associated with an increased risk of AD. No association found between midlife diastolic hypertension and AD
Li (2019)	HBP HL	All-cause AD	11	Prospective cohort	Japan, Finland, Sweden, Greece, Italy, USA, Netherlands, Former Yugoslavia	Hypertension and hypercholesterolaemia were found to be associated with an increased risk of dementia, but results may be unreliable
Li (2020)	HF	All-cause AD	12	Longitudinal cohort and cross-sectional	Italy, France, Norway, USA, Germany, Sweden, Finland, Denmark, Netherlands	HF associated with an increased risk of both all-cause dementia and AD
Liang (2021)	CHD	AD VaD	28	Longitudinal cohort, case-control and cross-sectional	Finland, USA, Sweden, Netherlands, Italy, Denmark	CHD associated with an increased risk of VaD but not AD
Liu (2019)	AF	All-cause	11	Prospective cohort (includes 2 RCTs)	USA, UK, Sweden, Finland, Netherlands	AF was associated with an increased risk of dementia
Liu (2021)	Arterial stiffness	All-cause	5	Longitudinal cohort and cross-sectional	USA, Netherlands, Sweden, France	Arterial stiffness was associated with an increased risk of dementia when measured categorically, whilst continuous measures were not significantly associated
Meng (2014)	HBP HL	AD	9	Prospective and historical cohort	USA, Korea, Finland, Sweden	Hypertension and hypercholesterolaemia were associated with an increased risk of AD
Papanastasiou (2021)	AF	All-cause	43	Prospective and retrospective cohort, and cross sectional (includes 2 RCTs)	NR	AF associated with an increased risk of dementia
Power (2011)	HBP	AD	18	Prospective cohort and nested case-control	France, Italy, USA, Taiwan, China, Canada, Spain, Nigeria, Sweden	Hypertension was not associated with an increased risk of AD
Proietti (2020)	AF	AD	6	Prospective cohort (one study appears to be cross-sectional)	USA, Sweden, Finland	AF was associated with an increased risk of AD
Santangeli (2012)	AF	All-cause	8	Prospective cohort (includes 2 RCTs)	Finland, USA, Sweden, Italy, UK, Romania, China, Russia, Bulgaria	AF was associated with an increased risk of all-cause dementia
Sharp (2011)	HBP	VaD	11	Longitudinal cohort and cross-sectional	USA, Taiwan, Canada	Hypertension was associated with an increased risk of VaD
Tang (2019)	HL	AD	27	Case-control studies	China, Japan, USA, India, Switzerland, Turkey, Germany, Portugal, Netherlands, Germany, Spain, Italy, Iran	High TC in late-life associated with an increased risk of future AD
Wang (2018)	HBP	All-cause	17	Prospective cohort and nested case-control	Sweden, Nigeria, USA, UK, Japan, South Korea, Finland	The association between hypertension and dementia varies depending on age, with an inverse associated more often observed in late-life
Wolters (2018)	CHD HF	All-cause AD	23	Longitudinal and prospective cohort	USA, Norway, Sweden, Italy, Netherlands, China, Finland, Taiwan, Poland, Denmark	CHD and HF were associated with an increased risk of dementia
Wu (2019)	HL	AD	37	Longitudinal and case-control	India, Japan, Turkey, China, Iran	TC and LDL-C associated with an increased risk of AD in Asian populations
Xu (2015)	HF AF HBP HL	AD	351	Longitudinal cohort and retrospective case-control	NR	HF, AF, hypertension, TC and HDL-C were not associated with an increased risk of AD. However, high systolic hypertension was associated with an increased risk of AD
Zhou (2020)	HL	AD	26	Case control	Japan, Spain, Brazil, China, USA, Finland, Serbia, Australia, Itlay, Hungary, Poland, Jordan, Sweden, Turkey	LDL-C was associated with an increased risk of AD
Zuin (2021)	AF	All-cause AD	18	Longitudinal and retrospective cohort	South Korea, Canada, UK, Netherlands, Taiwan, USA, Sweden, Finland, Italy	AF was associated with an increased risk of both all-cause dementia and AD

AD, Alzheimer's disease; AF, atrial fibrillation; CHD, coronary heart disease; HBP, high blood pressure; HDL-C, high-density lipoprotein cholesterol; HF, heart failure; HL hyperlipidaemia; LDL-C, low-density lipoprotein cholesterol; NR, not reported; RCT, randomised control trial; TC, total cholesterol; VaD, vascular dementia.

The methodological quality of the included reviews had a median score of 83% on the JBI critical appraisal tool for systematic reviews and research syntheses (range 3–11) (26). Twenty-three of the included reviews achieved ≥60%, indicating good methodological quality. The most neglected items were using appropriate critical appraisal (item 5; 31%), using two reviewers for this process (item 6, 51%), minimizing errors in data extraction (item 7; 49%), and recommending policy/practice changes in line with the data (item 10; 31%).

### Coronary heart disease

**Figure 2:**
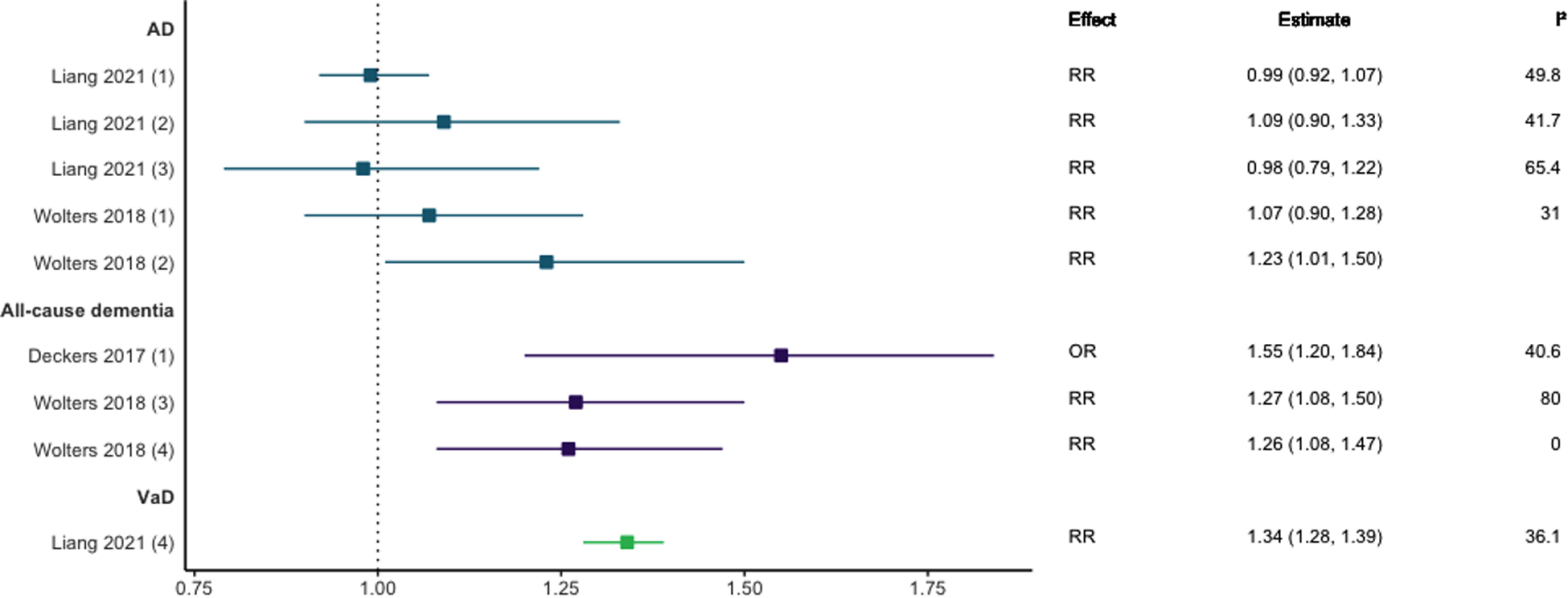
Effect estimates and 95% confidence intervals of meta-analyses reporting an association between CHD and dementia, with the vertical line representing no effect. AD, Alzheimer's disease; CHD, coronary heart disease; OR, odds ratio; RR, risk ratio; VaD, vascular dementia.

Three meta-analyses investigated the association between CHD and dementia, with mixed results as shown in Figure 2 (8, 10, 27). Two meta-analyses including prospective and case-control or longitudinal cohort designs focusing on CHD and all-cause dementia reported a strong positive association [RR = 1.26, 95% CI; 1.08–1.50 (10) and OR = 1.55, 95% CI; 1.20–1.84 (8)]. Regarding dementia subtypes, meta-analyses investigating the relationship between AD and CHD [including when myocardial infarction (MI) and angina pectoris (AP) were analysed separately] found no significant associations overall (10, 27) and a significant association in population-based AD studies only (RR = 1.23, 95% CI: 1.01–1.50) (10). In contrast, CHD was significantly associated with VaD in included longitudinal cohort and case-control studies (RR = 1.34, 95% CI; 1.28–1.39) (27).

### Heart failure

**Figure 3:**
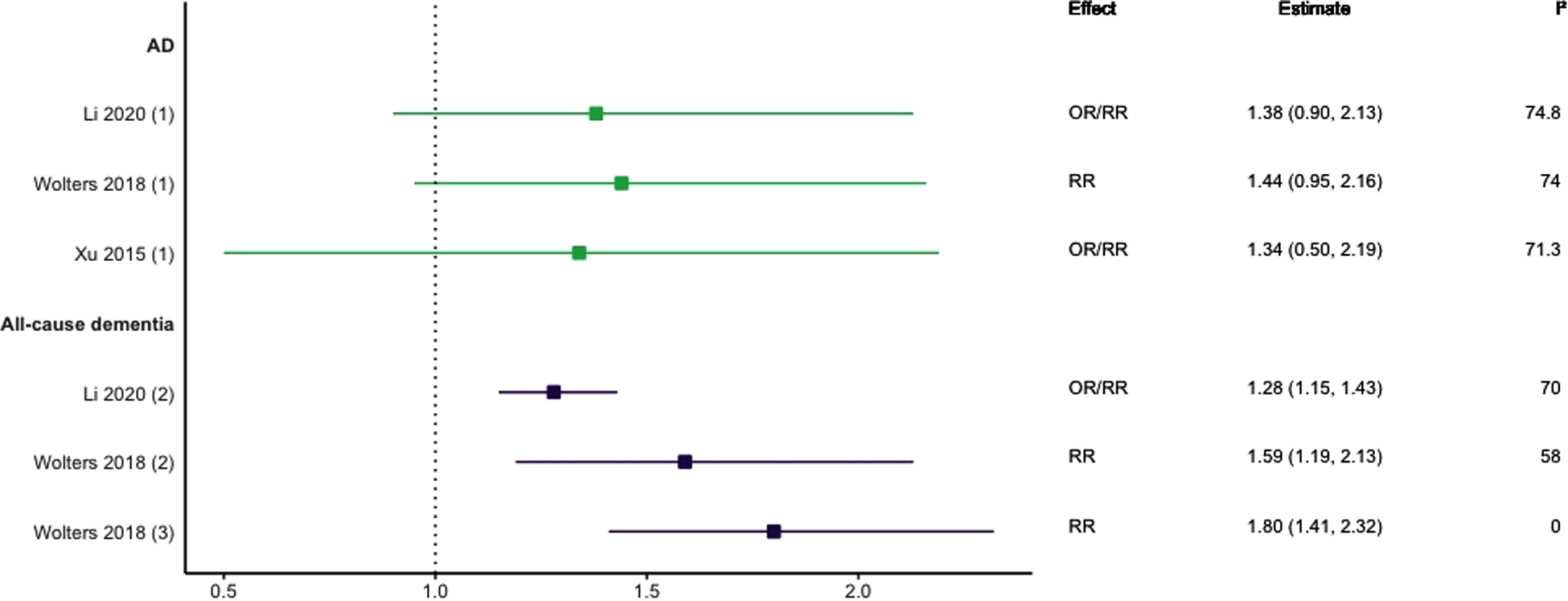
Effect estimates of meta-analyses reporting an association between heart failure and dementia, with the vertical line representing no effect. To note, Xu et al. treated ORs as approximates to RRs, as such, violations of this assumption may have implications for the comparability of ORs and RRs, meaning that interpretation of these pooled estimates should be done cautiously. AD, Alzheimer's disease; OR, odds ratio; RR, risk ratio.

Three meta-analyses investigating HF and dementia were identified (10, 28, 29). As shown in Figure 3, those studies focused on all-cause dementia reported significant positive associations with effect sizes ranging from 1.28 (95% CI; 1.15–1.43) to 1.80 (95% CI; 1.41–2.32) (10, 28). For studies focusing solely on AD, there were no statistically significant associations across longitudinal cohort and case-control designs (10, 28, 29).

### Atrial fibrillation

**Figure 4:**
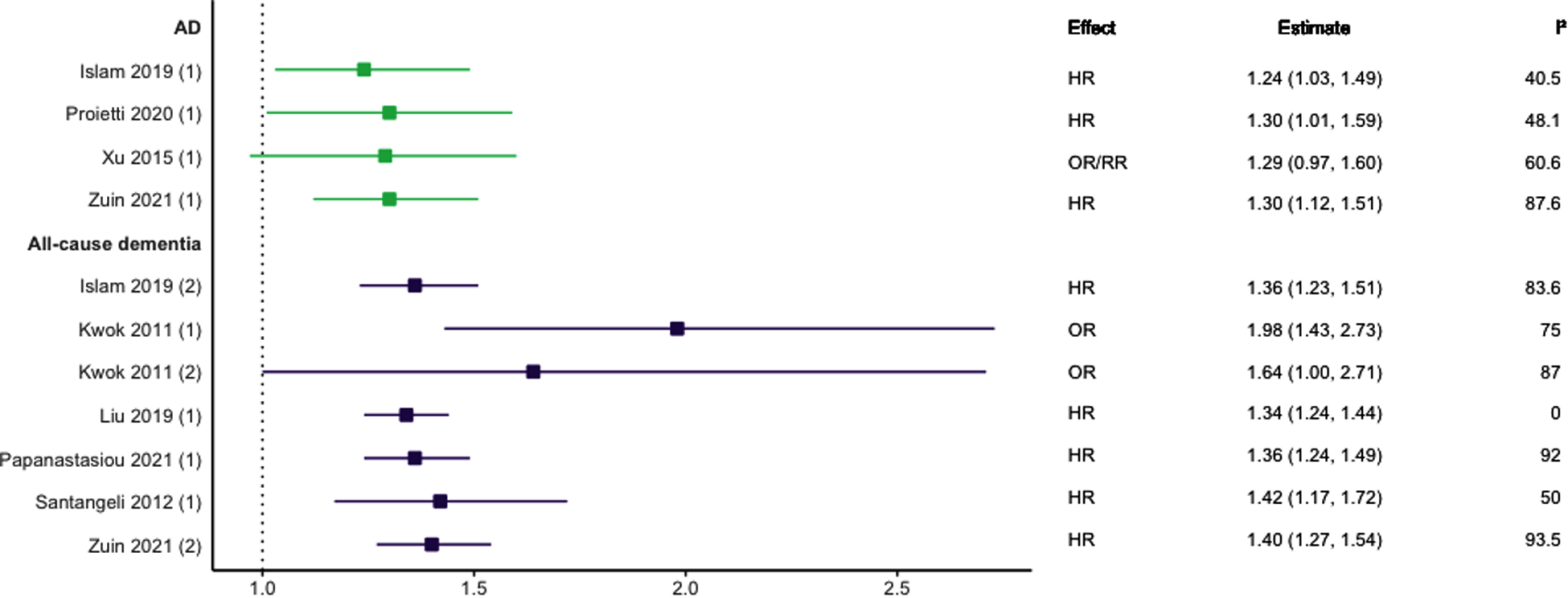
Effect estimates of meta-analyses reporting an association between atrial fibrillation and dementia, with the vertical line representing no effect. To note, Xu et al. treated ORs as approximates to RRs, as such, violations of this assumption may have implications for the comparability of ORs and RRs, meaning that interpretation of these pooled estimates should be done cautiously. AD, Alzheimer's disease; HR, hazard ratio; OR, odds ratio; RR, risk ratio.

Eight meta-analyses focused on AF (29–36). This included five studies focused on prospective cohort designs (31–35), one on longitudinal cohort designs (36), and two focused on both longitudinal cohort and retrospective case-control study designs (29, 30). Four studies also included one or two individual studies that utilised secondary data from randomised control trials (30–32, 35). As shown in Figure 4, there was consensus supporting a positive association between AF and all-cause dementia (30–33, 35, 36), AD (30, 33, 34, 36), and VaD (33); with only one meta-analysis not finding a statistically significant association between AF and AD (29).

### Hypertension

**Figure 5:**
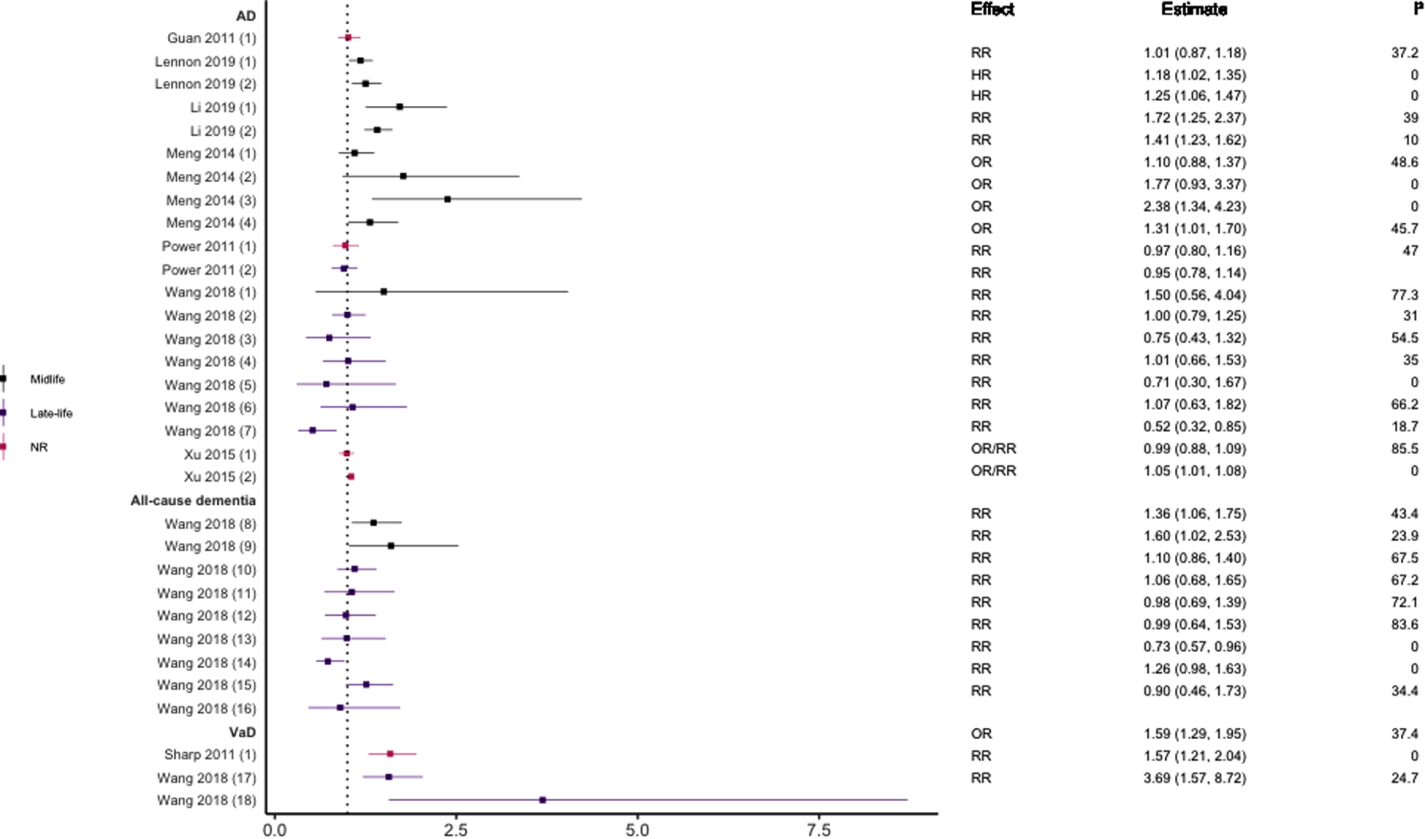
Effect estimates of meta-analyses reporting an association between hypertension (various definitions) and dementia. The vertical line represents no effect, with distinct colours conveying age stratification. To note, Xu et al. treated ORs as approximates to RRs, as such, violations of this assumption may have implications for the comparability of ORs and RRs, meaning that interpretation of these pooled estimates should be done cautiously. AD, Alzheimer's disease; HR, hazard ratio; NR, not reported; OR, odds ratio; RR, risk ratio; VaD, vascular dementia.

Eight meta-analyses (29, 37–43) reported on hypertension and the risk of all-cause dementia and its subtypes. There was wide variability in the definition of hypertension and dementia outcomes used. Of the eight reviews, two included longitudinal cohort studies only (37, 42); one prospective cohort studies only (39); one longitudinal and nested case-control designs (38); one both prospective and historical cohort designs (40); two included prospective and nested case-control designs (41, 43); and one included longitudinal and retrospective cohort designs (29). See Figure 5 for the results of the meta-analyses.

Two studies did not find a significant association between hypertension and AD (29, 37) while three studies did including increased risk for of all-cause dementia (43), VaD (42), and AD (29). There was some evidence suggesting that the magnitude and direction of associations may differ by age (younger), dementia subtype (VaD), and metric of blood pressure (SBP). For example, Wang et al. reported that the risk of all-cause dementia differs across the life course, finding that individuals aged <65 years were at higher risk of all-cause dementia in late-life if they had hypertensive SBP or diastolic blood pressure (DBP) in midlife (43). This was not the case in those aged >65 years in which no statistically significant association was found for higher SBP, with higher DBP being protective. They also found no significant association between SBP and AD in all age groups, but that higher DBP was protective in those aged 75–85 years. This latter finding supported in another review (41).

Some meta-analyses focused specifically on the link between midlife hypertension and risk of dementia. Definitions of midlife did however differ, capturing individuals ≤60 (38), 35–65 (39) or 40–65 years (40). Two reviews (38, 39) divided hypertension into borderline hypertension (SBP ≤140 mmHg) and clinical hypertension (SBP ≤160 mmHg). Borderline and clinical SBP during midlife was associated with an increased risk of developing AD and all-cause dementia in later life. In line with these findings, Meng et al., reported an overall significant association between midlife hypertension and AD (40). This association remained when DBP was analysed separately but was not significant for SBP. Moreover, incremental increases in both SBP and DBP were not significantly associated with increased risk of AD in the individual studies reviewed by Lennon and colleagues (38).

### Hyperlipidaemia

**Figure 6:**
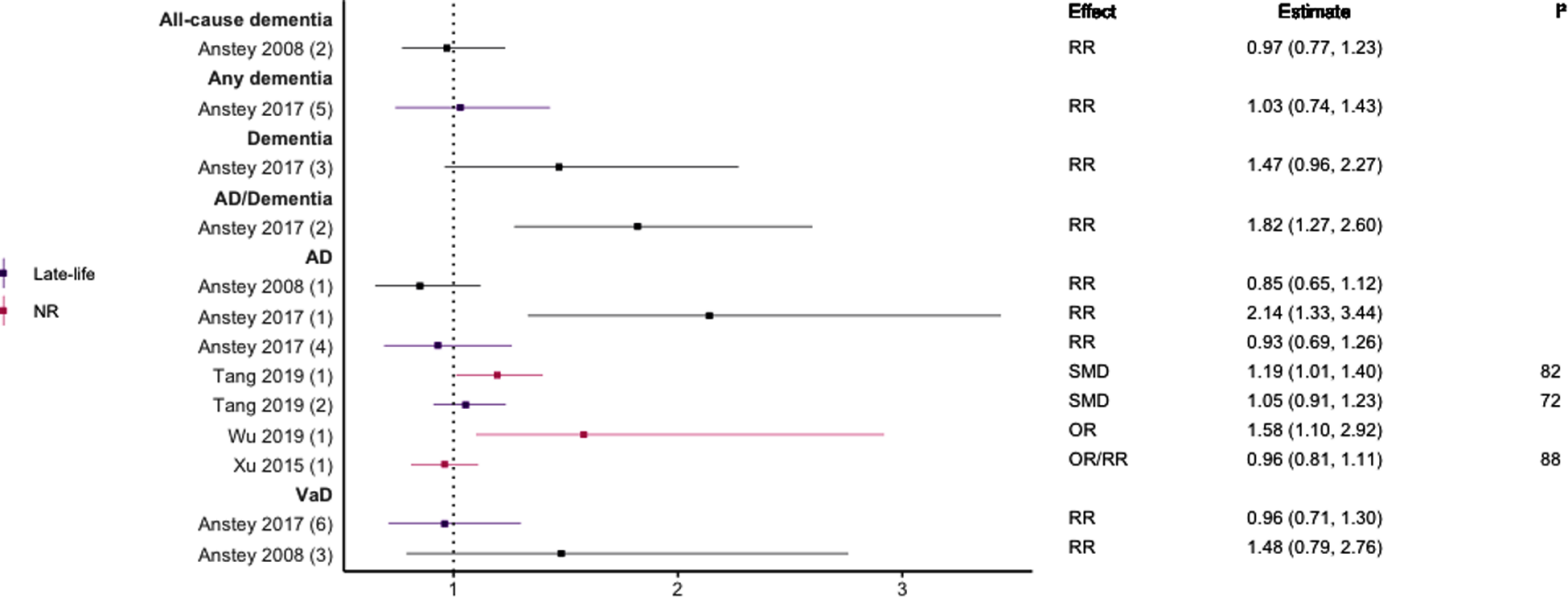
Effect estimates of meta-analyses reporting an association between TC and dementia. The vertical line represents no effect, with different colours conveying age stratification. To note, Xu et al. treated ORs as approximates to RRs, as such, violations of this assumption may have implications for the comparability of ORs and RRs, meaning that interpretation of these pooled estimates should be done cautiously. Moreover, SMDs have been converted to approximate ORs using $OR=exp\left(\frac{\pi }{3}\times SMD\right)$. AD, Alzheimer's disease; NR, not reported; OR, odds ratio; RR, risk ratio; SMD, standard mean difference; VaD, vascular dementia.

**Figure 7:**
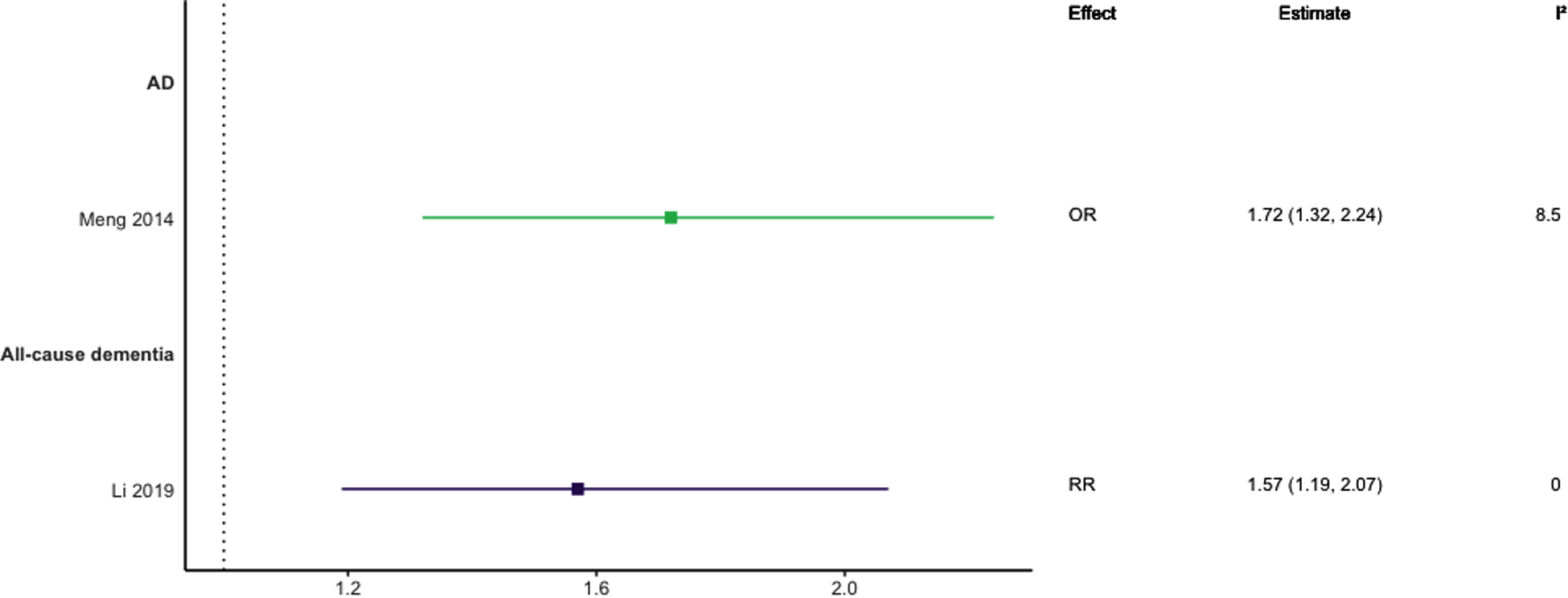
Effect estimates of meta-analyses reporting an association between hypercholesterolaemia and dementia, with the vertical line representing no effect. AD, Alzheimer's disease; OR, odds ratio; RR, risk ratio.

**Figure 8:**
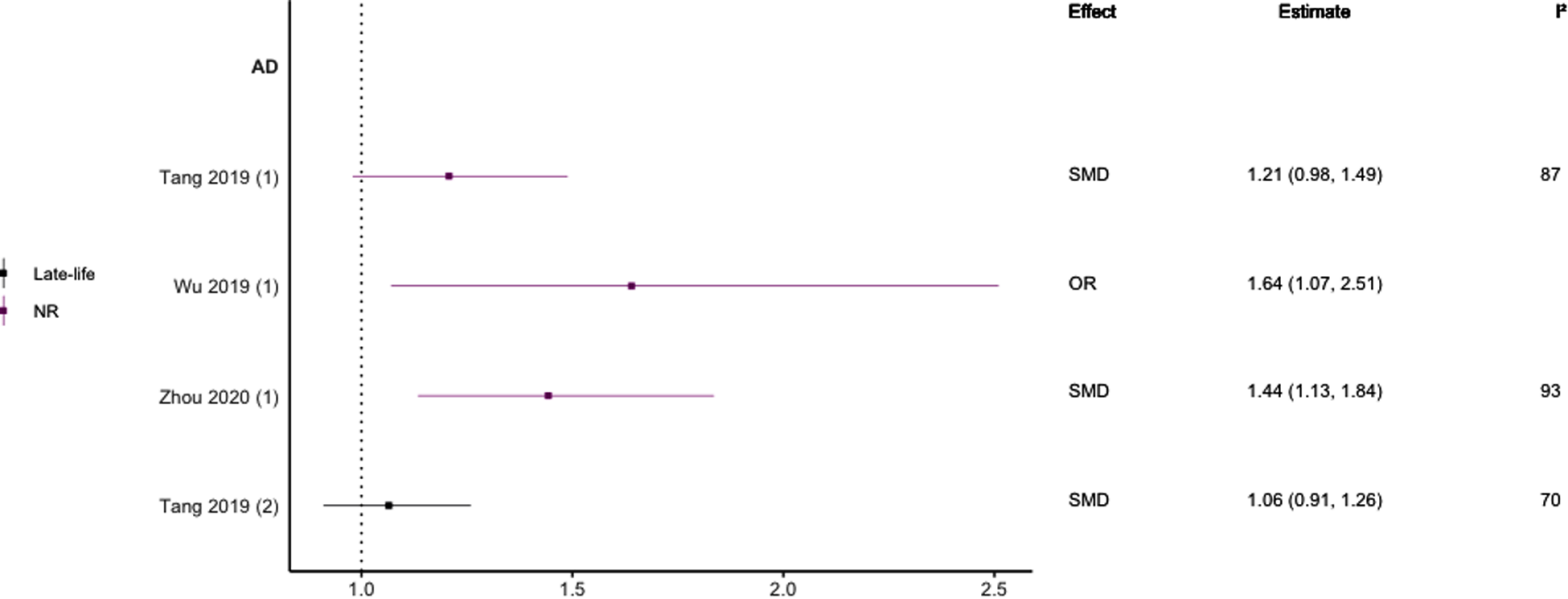
Effect estimates of meta-analyses reporting an association between LDL-C and AD. The vertical line represents no effect, with different colours conveying age stratification. Note, SMDs have been converted to approximate ORs using $OR=exp\left(\frac{\pi }{3}\times SMD\right)$. AD, Alzheimer's disease; OR, odds ratio; SMD, standard mean difference.

**Figure 9:**
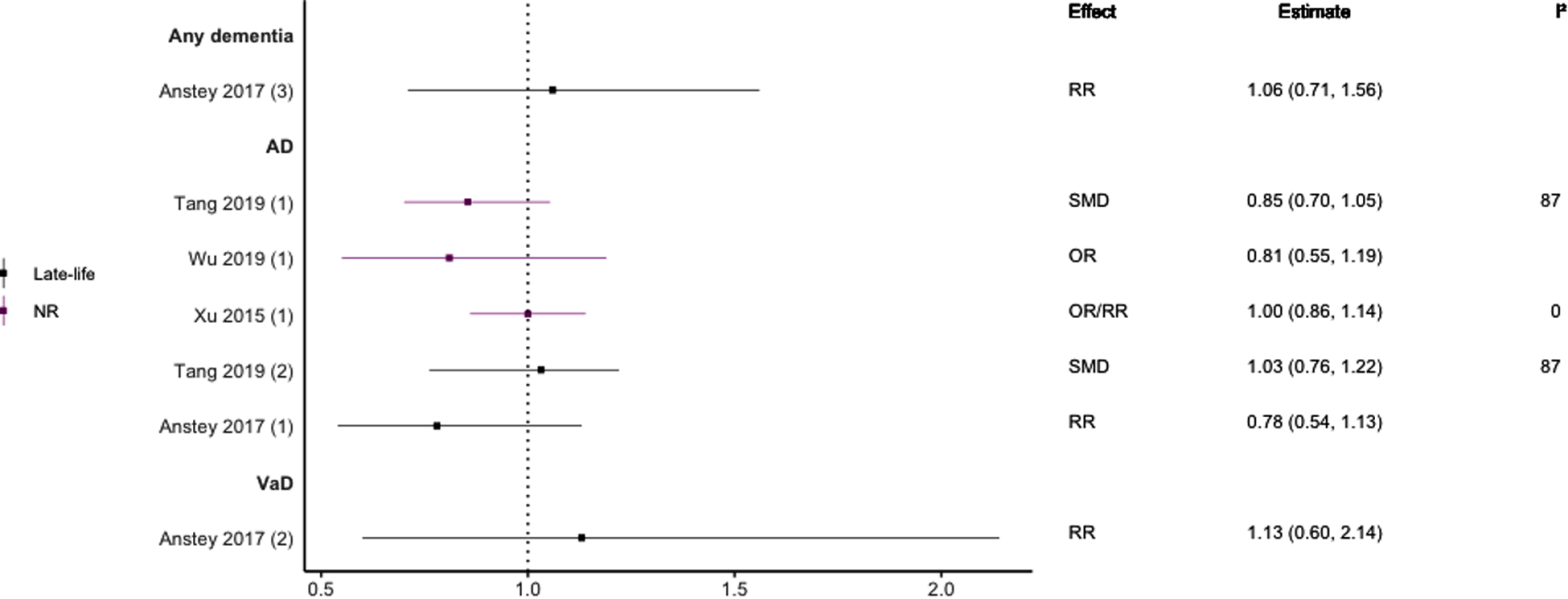
Effect estimates of meta-analyses reporting an association between HDL-C and dementia. The vertical line represents no effect, with different colours conveying age stratification. To note, Xu et al. treated ORs as approximates to RRs, as such, violations of this assumption may have implications for the comparability of ORs and RRs, meaning that interpretation of these pooled estimates should be done cautiously. Moreover, SMDs have been converted to approximate ORs using $OR=exp\left(\frac{\pi }{3}\times SMD\right)$. AD, Alzheimer's disease; OR, odds ratio; RR, risk ratio; SMD, standard mean difference; VaD, vascular dementia.

**Figure 10:**
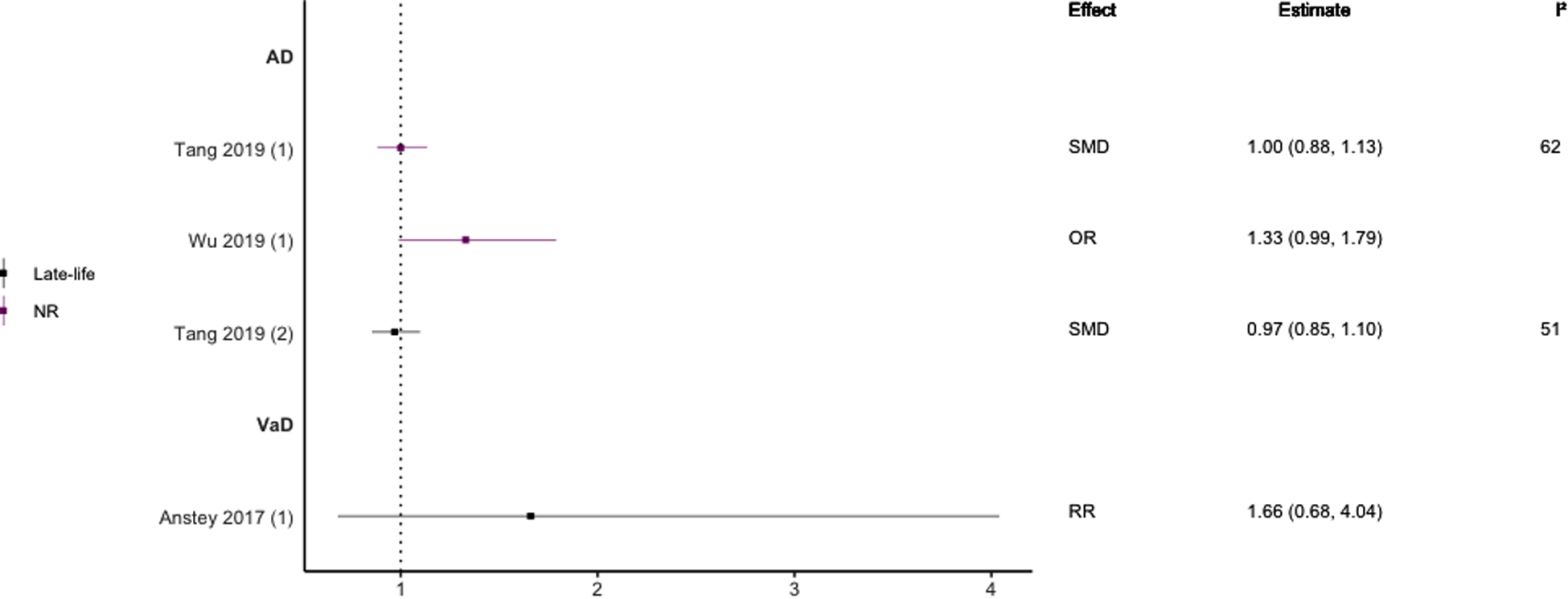
Effect estimates of meta-analyses reporting an association between TG and dementia. The vertical line represents no effect, with different colours conveying age. Note, SMDs have been converted to approximate ORs using $OR=exp\left(\frac{\pi }{3}\times SMD\right)$. AD, Alzheimer's disease; OR, odds ratio; RR, risk ratio; SMD, standard mean difference; VaD, vascular dementia.

Eight meta-analyses focused on hyperlipidaemia (29, 39, 40, 44–48). Three included prospective cohort designs only (39, 44, 45), two studies included only case-control designs (46, 48), one included three included longitudinal cohort or retrospective case-control designs (29), one included prospective and historical cohort designs (40), and one included case-control and longitudinal cohort designs (47). Seven meta-analyses investigated TC or hypercholesterolemia, three LDL-C, four HDL-C, and three TG (see Figures 6–10). Definitions of these measurements differed between studies, for example, some studies defined high TC levels as the mean difference in TC levels between cases and non-cases, whilst another used quartiles.

In general, findings were mixed. Two meta-analyses found a significant positive association between high TC levels and risk of all-cause dementia (39, 44), whilst two others found no association with all-cause dementia (45) or all-cause dementia plus AD (44). No association was found between high levels of TC in late-life and all-cause dementia (44). Two of three reviews found a significant, positive association between high TC levels and AD without stratifying results by age (SMD = 0.17, 95% CI; 0.01–0.32), with one of these studies focused specifically on Asian populations (OR = 1.58, 95% CI; 1.10–2.92); both reported substantial heterogeneity. Others found no association between high TC and AD (29). A diagnosis of hypercholesterolemia in midlife was found to be significantly associated with an increased risk of AD (Combined OR = 1.72, 95% CI; 1.32–2.24), and an updated review comparing the highest and lowest quartiles of TC levels in midlife found a two-fold increase of AD risk (RR 2.14; 95% CI 1.33–3.44). No association was found between late-life high TC/hypercholesterolemia and AD (44, 46), or VaD (44).

Regarding HDL-C, no significant association was found with risk of all-cause dementia in late-life (44), and AD without age stratification (29, 46, 47). HDL-C when measured in late-life was not significantly associated with the risk of developing AD (44, 46) or VaD (44). One subgroup meta-analysis of those aged <70 years found high levels of HDL-C to be significantly protective of future AD (SMD = −0.50, 95% CI; −0.76 to −0.25). Without age-stratifying analyses, two studies found an association between high LDL-C levels and AD (47, 48). One study found no association between high LDL-C levels in late-life/no age stratification and AD (46). Late-life measures of TG were not associated with either AD (46) or VaD (44), as well as when results were not age stratified with future AD (46, 47).

### Arterial stiffness

Only one meta-analysis was identified that examined the relationship between arterial stiffness and the risk of dementia, specifically measuring aortic stiffness by aortic pulse wave velocity (PWV) (49). When measured categorically (low vs. high) aortic stiffness was associated with a two-fold increased risk of incident dementia in longitudinal studies (OR = 2.10, 95% CI; 1.16–3.80). However, when aortic stiffness was measured continuously, this association was no longer significant (OR = 1.11, 95% CI; 0.98–1.25). There was some suggestion that age may increase the risk of dementia and/or cognitive impairment caused by higher aortic PWV.

## Discussion

### Main findings

This is the first umbrella review to synthesise the meta-analytic evidence on the association between incident dementia and CHD, HF, AF, hypertension, hyperlipidaemia, and arterial stiffness. The findings highlight the strongest evidence for increased dementia risk from CHD, HF, AF, and midlife hypertension. There is limited evidence on arterial stiffness.

Most studies that investigated CHD, HF, and AF found strong and consistent associations with all-cause dementia and VaD, whilst results concerning AD varied. Potential mechanisms underlying these three conditions include reduced cardiac output and impaired haemodynamic responses which can lead to cerebral hypoperfusion and hypoxia (50), systemic inflammation, resulting in cerebral small vessel disease (51). However, these mechanisms are possibly bidirectional. For example, it cannot be ruled out that dysregulation in the electrical conduction of the atrium occurs in response to neurodegenerative diseases (52).

Regarding hypertension, the pattern of results was mixed likely due to heterogeneity in the definitions of hypertension used, time of testing/disease duration (e.g., mid-life vs. later life), treatment status (e.g., use of antihypertensives vs. no treatment), varied length of follow-up, and dementia outcome tested. However, it is important to note that all pooled estimates measuring midlife hypertension and the risk of all-cause dementia found a significant positive association, whether measured by SBP or DBP. However, the only meta-analysis looking at the relationship between hypertension in late-life and all-cause dementia found no significant association when analysing SBP and DBP separately or together. Regarding AD, findings were mixed with some studies reporting a significant positive association between midlife hypertension and AD and others not. Studies measuring hypertension in late-life or across the entire life course and the risk of AD found either no significant association or an inverse significant (protective) association. All meta-analyses investigating hypertension and VaD found significant associations, with these studies either measuring hypertension in late-life or having no age restrictions at baseline.

Chronic hypertension in midlife has been associated with reduced white matter integrity and impaired cerebral autoregulation (through reduced arterial elasticity for example), all of which have been linked to dementia (53). In contrast, in late-life, higher blood pressure may compensate for age-associated vascular changes such as increased vascular stiffness and endothelial dysfunction (54), helping maintain adequate cerebral perfusion and therefore being linked to decreased dementia risk (55). The non-significant and inverse results concerning late-life hypertension in all-cause dementia and AD may be explained by the age-related decline in blood pressure and the compensatory mechanisms hypertension may hold in late-life.

Results were often unclear and inconsistent regarding hyperlipidaemia. High TC or a diagnosis of hypercholesterolemia were more consistently associated with dementia as compared to other measures of hyperlipidaemia. More specifically, midlife measures of high TC were more consistently associated with all-cause dementia and AD compared to results either not stratified by age or in late-life. Although no meta-analyses found an inverse association between TC levels and dementia, two primary studies identified in one systematic review found high TC levels in late-life to decrease the risk of future dementia (56). Studies focusing on HDL-C showed either no association with dementia, or that it was significantly protective in the case of all-cause dementia and AD, although these studies either focused on late-life cholesterol measures or did not age stratify their results. Although few studies focused on LDL-C measurements and dementia, significant associations were only found in two analyses that did not take into account age groups and focused only on AD, and in both of which substantial heterogeneity was observed. Furthermore, one analysis found no significant association between LDL-C and AD in late-life cohorts. No included study or stated primary study found an association between TG and all-cause dementia, AD, or VaD in late-life cohorts or not age restricted, with no meta-analysis conducted in midlife cohorts.

There are a number of possible mechanisms linking cholesterol and dementia. Some evidence suggests that lipids in the brain may affect cerebral enzyme functioning that protects against the formation of insoluble Aβ proteins and oxidative stress. However, lipids in the blood may not be directly transported into the brain and nervous system as prevented by the blood-brain barrier (13), making it harder to ascertain positive associations between blood lipid levels and dementia. This complex interaction may be amplified by other factors such as lifestyle and genetics that interact with lipid metabolism, such as the ApoE ɛ4 genotype helping mediate brain lipid transportation and metabolism (57). Moreover, the inverse associations more frequently reported in late-life may reflect the observed physical and neurodegenerative changes preceding dementia diagnosis, such as weight loss, which may alter cholesterol levels (56, 58). Regarding the more consistent results shown in measures of cholesterol in midlife, high levels at this time point may influence the development of dementia pathology, such as increasing oxidative stress and the build-up of Aβ (44).

Concerning arterial stiffness, the findings from one review suggests an increased risk of all-cause dementia. Arterial stiffness has been associated with structural brain changes and cerebral microvascular damage which may ultimately lead to dementia as a consequence of the excessive pulsative pressure from the main arteries (59). Moreover, arterial stiffness is often associated with other CVDs linked to dementia, including hypertension and atherosclerosis, although it is unknown whether arterial stiffness precedes these manifestations or is consequential of them (60).

### Strengths and weaknesses

This review has numerous strengths. The search strategy was comprehensive and utilised wide search terms and definitions of CVD. Moreover, all key dementia outcomes (e.g., all-cause, AD and VaD) were included. This enabled a comprehensive summary of the associations of multiple CVDs with all-cause dementia and its most prevalent sub-types. Further, 91% of the included reviews had good methodological quality as rated by the JBI Critical Appraisal Checklist. However, as previously mentioned, numerous methodological limitations were not always reflected in the critical appraisal scores, which questions the sensitivity of these tools and flags the necessity of iterative quality improvements to such tools informed by systematic and umbrella reviews such as this. This umbrella review also followed a systematic and methodological pre-registered approach based on best practice guidelines on conducting reviews of this kind. There are however some limitations. First, our review relied on published systematic reviews and meta-analyses and therefore is limited by the quality and comprehensiveness of these. Indeed, key primary studies may have been missed. Second, even across reviews focusing on the same CVD condition, there was large heterogeneity in the types of studies included (e.g., cohort, case-control, randomised-control trial), definitions of disease, operationalisation of dementia (and AD/VaD), timing of risk factor assessment (mid vs. late life), control of confounding factors and sensitivity analysis; all of which can affect comparability. Moreover, we found meta-analyses combining different study designs and types of effect estimates (hazard ratios, risk ratios and odds ratios), as well as inconsistency in the use and reporting of unadjusted and adjusted covariates. One illustration comes from Xu and colleagues who treated ORs as approximates of RRs. We would like to draw the reader's attention to the fact that ORs will only mirror RRs if the occurrence of the outcome is uncommon across a subset of the population. If the outcome is frequent, then ORs can greatly overestimate the RR and estimates on the OR scale may impact the pooled estimate. Lastly, we restricted our search to reviews published in English. All eligible reviews incorporated studies from populations exclusively in North America and Europe with a paucity of studies from LMICs. While this restriction in geographical location could be due to the language criteria, it is important to note that to date very little dementia research has been conducted in LMICs. This is a notable gap and key research priority (61).

### Summary and implications

The findings highlight that interventions targeting CHD, HF, AF, arterial stiffness, and midlife hypertension could make a significant impact on dementia numbers, particularly VaD and all-cause dementia. More work is urgently needed to understand the mechanistic link between heart and brain health and develop intervention strategies to concurrently impact incidence of these diseases. This is particularly important in LMICs where research is scare yet the prevalence of both CVD and dementia is rising.
