# Supplementary material for: Cardiovascular disease, associated risk factors, and risk of dementia: An umbrella review of meta-analyses
Source: Front Epidemiol. 2023 Feb 9;3:1095236. doi: 10.3389/fepid.2023.1095236 (PMC10910908; doi:10.3389/fepid.2023.1095236)
Supplement: Supplementary file 1 [file Datasheet1.pdf]

Supplementary Material

Supplementary Table 1. Search strategy for each database searched

Database(s): **Ovid MEDLINE(R) ALL** 1946 to October 06, 2021
Search Strategy:

#	Searches	Results
1	exp Dementia/	180977
2	(dement* or Alzheim*).mp. [mp=title, abstract, original title, name of substance word, subject heading word, floating sub-heading word, keyword heading word, organism supplementary concept word, protocol supplementary concept word, rare disease supplementary concept word, unique identifier, synonyms]	266433
3	1 or 2	284820
4	exp Heart Failure/	131469
5	((myocard* or cardia* or heart or ventric*) adj (failure or decompensation or insufficien* or dysfunction*)).mp. [mp=title, abstract, original title, name of substance word, subject heading word, floating sub-heading word, keyword heading word, organism supplementary concept word, protocol supplementary concept word, rare disease supplementary concept word, unique identifier, synonyms]	280846
6	cardiomyopath*.mp. [mp=title, abstract, original title, name of substance word, subject heading word, floating sub-heading word, keyword heading word, organism supplementary concept word, protocol supplementary concept word, rare disease supplementary concept word, unique identifier, synonyms]	105086
7	4 or 5 or 6	351903
8	exp Hypertension/	299274

9	(hypertens* or "blood pressure").mp. [mp=title, abstract, original title, name of substance word, subject heading word, floating sub-heading word, keyword heading word, organism supplementary concept word, protocol supplementary concept word, rare disease supplementary concept word, unique identifier, synonyms]	837454
10	exp Blood Pressure Monitoring, Ambulatory/	10660
11	("24-hour blood pressure monitoring" or "home blood pressure monitoring").mp. [mp=title, abstract, original title, name of substance word, subject heading word, floating sub-heading word, keyword heading word, organism supplementary concept word, protocol supplementary concept word, rare disease supplementary concept word, unique identifier, synonyms]	874
12	8 or 9 or 10 or 11	837478
13	("atrial fibrillation" or "atrial flutter" or AFIB or arrhythmi* or AF).mp. [mp=title, abstract, original title, name of substance word, subject heading word, floating sub-heading word, keyword heading word, organism supplementary concept word, protocol supplementary concept word, rare disease supplementary concept word, unique identifier, synonyms]	251951
14	exp Arrhythmias, Cardiac/	221098
15	13 or 14	324698
16	exp Vascular Stiffness/	7068
17	("arterial stiffness" or "pulse pressure" or "wave reflections" or "augmentation index" or "pulse wave velocity" or "aortic stiffness" or "central pressure" or "arterial compliance").mp. [mp=title, abstract, original title, name of substance word, subject heading word, floating sub-heading word, keyword heading word, organism supplementary concept word, protocol supplementary concept word, rare disease supplementary concept word, unique identifier, synonyms]	25082
18	16 or 17	26141
19	exp Myocardial Ischemia/	447384
20	(((isch?emic or coronary) and (heart or arter* or disease or syndrome)) or myocard* or angina).mp. [mp=title, abstract, original title, name of substance word, subject heading word, floating sub-heading word, keyword heading word, organism supplementary concept word, protocol supplementary concept word, rare disease supplementary concept word, unique identifier, synonyms]	1010859

21	19 or 20	1018728
22	exp Hyperlipidemias/	68030
23	(hyperlipid?emia or dyslipid?emia or hypercholesterol?emia or cholesterol or HDL or LDL).mp. [mp=title, abstract, original title, name of substance word, subject heading word, floating sub-heading word, keyword heading word, organism supplementary concept word, protocol supplementary concept word, rare disease supplementary concept word, unique identifier, synonyms]	396380
24	22 or 23	409682
25	7 or 12 or 15 or 18 or 21 or 24	2368185
26	(systematic review or meta-analysis).pt.	240852
27	meta-analysis/ or systematic review/ or systematic reviews as topic/ or meta-analysis as topic/ or "meta analysis (topic)"/ or "systematic review (topic)"/ or exp technology assessment, biomedical/ or network meta-analysis/	274471
28	((systematic* adj3 (review* or overview*)) or (methodologic* adj3 (review* or overview*))).ti,ab,kf,kw.	242670
29	((quantitative adj3 (review* or overview* or synthes*)) or (research adj3 (integrati* or overview*))).ti,ab,kf,kw.	12798
30	((integrative adj3 (review* or overview*)) or (collaborative adj3 (review* or overview*)) or (pool* adj3 analy*)).ti,ab,kf,kw.	31976
31	(data synthes* or data extraction* or data abstraction*).ti,ab,kf,kw.	32342
32	(handsearch* or hand search*).ti,ab,kf,kw.	10073
33	(mantel haenszel or peto or der simonian or dersimonian or fixed effect* or latin square*).ti,ab,kf,kw.	30148
34	(met analy* or metanaly* or technology assessment* or HTA or HTAs or technology overview* or technology appraisal*).ti,ab,kf,kw.	10518
35	(meta regression* or metaregression*).ti,ab,kf,kw.	11256
36	(meta-analy* or metaanaly* or systematic review* or biomedical technology assessment* or bio-medical technology assessment*).mp,hw.	372788
37	(medline or cochrane or pubmed or medlars or embase or cinahl).ti,ab,hw.	269834

38	(cochrane or (health adj2 technology assessment) or evidence report).jw.	20393
39	(comparative adj3 (efficacy or effectiveness)).ti,ab,kf,kw.	15194
40	(outcomes research or relative effectiveness).ti,ab,kf,kw.	10231
41	((indirect or indirect treatment or mixed-treatment or bayesian) adj3 comparison*).ti,ab,kf,kw.	3725
42	(meta-analysis or systematic review).mp.	348274
43	(multi* adj3 treatment adj3 comparison*).ti,ab,kf,kw.	263
44	(mixed adj3 treatment adj3 (meta-analy* or metaanaly*)).ti,ab,kf,kw.	174
45	umbrella review*.ti,ab,kf,kw.	747
46	(multi* adj2 paramet* adj2 evidence adj2 synthesis).ti,ab,kf,kw.	13
47	(multiparamet* adj2 evidence adj2 synthesis).ti,ab,kf,kw.	17
48	(multi-paramet* adj2 evidence adj2 synthesis).ti,ab,kf,kw.	11
49	or/26-48	557344
50	3 and 25 and 49	850

Database(s): **Embase** 1974 to 2021 Week 39
Search Strategy:

#	Searches	Results
1	exp dementia/	388698
2	(dement* or Alzheim*).mp. [mp=title, abstract, heading word, drug trade name, original title, device manufacturer, drug manufacturer, device trade name, keyword heading word, floating subheading word, candidate term word]	400611
3	1 or 2	457053
4	exp heart failure/	548773

5	((myocard* or cardia* or heart or ventric*) adj (failure or decompensation or insufficien* or dysfunction*)).mp. [mp=title, abstract, heading word, drug trade name, original title, device manufacturer, drug manufacturer, device trade name, keyword heading word, floating subheading word, candidate term word]	493780
6	cardiomyopath*.mp. [mp=title, abstract, heading word, drug trade name, original title, device manufacturer, drug manufacturer, device trade name, keyword heading word, floating subheading word, candidate term word]	169787
7	4 or 5 or 6	726385
8	exp hypertension/	791905
9	(hypertens* or "blood pressure").mp.	1369928
10	exp blood pressure monitoring/	51951
11	("24-hour blood pressure monitoring" or "home blood pressure monitoring").mp. [mp=title, abstract, heading word, drug trade name, original title, device manufacturer, drug manufacturer, device trade name, keyword heading word, floating subheading word, candidate term word]	1545
12	8 or 9 or 10 or 11	1424777
13	("atrial fibrillation" or "atrial flutter" or AFIB or arrhythmi* or AF).mp. [mp=title, abstract, heading word, drug trade name, original title, device manufacturer, drug manufacturer, device trade name, keyword heading word, floating subheading word, candidate term word]	393024
14	exp atrial fibrillation/ or exp heart atrium arrhythmia/ or exp heart fibrillation/	186949
15	13 or 14	443093
16	exp arterial stiffness/	24207
17	("arterial stiffness" or "pulse pressure" or "wave reflections" or "augmentation index" or "pulse wave velocity" or "aortic stiffness" or "central pressure" or "arterial compliance").mp. [mp=title, abstract, heading word, drug trade name, original title, device manufacturer, drug manufacturer, device trade name, keyword heading word, floating subheading word, candidate term word]	51574
18	16 or 17	51574

19	exp ischemic heart disease/	703720
20	(((isch?emic or coronary) and (heart or arter* or disease or syndrome)) or myocard* or angina).mp. [mp=title, abstract, heading word, drug trade name, original title, device manufacturer, drug manufacturer, device trade name, keyword heading word, floating subheading word, candidate term word]	1350769
21	19 or 20	1424726
22	exp hyperlipidemia/	167922
23	(hyperlipid?emia or dyslipid?emia or hypercholesterol?emia or cholesterol or HDL or LDL).mp. [mp=title, abstract, heading word, drug trade name, original title, device manufacturer, drug manufacturer, device trade name, keyword heading word, floating subheading word, candidate term word]	644592
24	22 or 23	655347
25	7 or 12 or 15 or 18 or 21 or 24	3466543
26	"systematic review".mp. or meta analysis.pt. [mp=title, abstract, heading word, drug trade name, original title, device manufacturer, drug manufacturer, device trade name, keyword heading word, floating subheading word, candidate term word]	398491
27	meta-analysis/ or systematic review/ or systematic reviews as topic/ or meta-analysis as topic/ or "meta analysis (topic)"/ or "systematic review (topic)"/ or exp technology assessment, biomedical/ or network meta-analysis/	489189
28	((systematic* adj3 (review* or overview*)) or (methodologic* adj3 (review* or overview*))).ti,ab,kf,kw.	299531
29	((quantitative adj3 (review* or overview* or synthes*)) or (research adj3 (integrati* or overview*))).ti,ab,kf,kw.	15089
30	((integrative adj3 (review* or overview*)) or (collaborative adj3 (review* or overview*)) or (pool* adj3 analy*)).ti,ab,kf,kw.	45306
31	(data synthes* or data extraction* or data abstraction*).ti,ab,kf,kw.	39746
32	(handsearch* or hand search*).ti,ab,kf,kw.	12266
33	(mantel haenszel or peto or der simonian or dersimonian or fixed effect* or latin square*).ti,ab,kf,kw.	39904

34	(met analy* or metanaly* or technology assessment* or HTA or HTAs or technology overview* or technology appraisal*).ti,ab,kf,kw.	17057
35	(meta regression* or metaregression*).ti,ab,kf,kw.	13916
36	(meta-analy* or metaanaly* or systematic review* or biomedical technology assessment* or bio-medical technology assessment*).mp,hw.	587078
37	(medline or cochrane or pubmed or medlars or embase or cinahl).ti,ab,hw.	353233
38	(cochrane or (health adj2 technology assessment) or evidence report).jw.	28625
39	(comparative adj3 (efficacy or effectiveness)).ti,ab,kf,kw.	22179
40	(outcomes research or relative effectiveness).ti,ab,kf,kw.	14691
41	((indirect or indirect treatment or mixed-treatment or bayesian) adj3 comparison*).ti,ab,kf,kw.	6396
42	(meta-analysis or systematic review).mp.	552309
43	(multi* adj3 treatment adj3 comparison*).ti,ab,kf,kw.	380
44	(mixed adj3 treatment adj3 (meta-analy* or metaanaly*)).ti,ab,kf,kw.	252
45	umbrella review*.ti,ab,kf,kw.	783
46	(multi* adj2 paramet* adj2 evidence adj2 synthesis).ti,ab,kf,kw.	25
47	(multiparamet* adj2 evidence adj2 synthesis).ti,ab,kf,kw.	18
48	(multi-paramet* adj2 evidence adj2 synthesis).ti,ab,kf,kw.	20
49	or/26-48	806629
50	3 and 25 and 49	2622

APA PsycInfo 1806 to September Week 4 2021

#	Searches	Results
1	exp Dementia/	82794
2	(dement* or Alzheim*).mp. [mp=title, abstract, heading word, table of contents, key concepts, original title, tests & measures, mesh]	116403
3	1 or 2	116936
4	((myocard* or cardia* or heart or ventric*) adj (failure or decompensation or insufficien* or dysfunction*)).mp. [mp=title, abstract, heading word, table of contents, key concepts, original title, tests & measures, mesh]	4653
5	cardiomyopath*.mp. [mp=title, abstract, heading word, table of contents, key concepts, original title, tests & measures, mesh]	978
6	4 or 5	5367
7	exp Hypertension/	7693
8	(hypertens* or "blood pressure").mp. [mp=title, abstract, heading word, table of contents, key concepts, original title, tests & measures, mesh]	39062
9	("24-hour blood pressure monitoring" or "home blood pressure monitoring").mp. [mp=title, abstract, heading word, table of contents, key concepts, original title, tests & measures, mesh]	26
10	7 or 8 or 9	39062
11	exp "Arrhythmias (Heart)"/	2273
12	("atrial fibrillation" or "atrial flutter" or AFIB or arrhythmi* or AF).mp. [mp=title, abstract, heading word, table of contents, key concepts, original title, tests & measures, mesh]	6767
13	11 or 12	7336

14	("arterial stiffness" or "pulse pressure" or "wave reflections" or "augmentation index" or "pulse wave velocity" or "aortic stiffness" or "central pressure" or "arterial compliance").mp. [mp=title, abstract, heading word, table of contents, key concepts, original title, tests & measures, mesh]	692
15	exp Heart Disorders/	15105
16	(((isch?emic or coronary) and (heart or artery or arteries or disease or syndrome)) or myocard* or angina).mp. [mp=title, abstract, heading word, table of contents, key concepts, original title, tests & measures, mesh]	23253
17	15 or 16	30470
18	(hyperlipid?emia or dyslipid?emia or hypercholesterol?emia or cholesterol or HDL or LDL).mp. [mp=title, abstract, heading word, table of contents, key concepts, original title, tests & measures, mesh]	12096
19	6 or 10 or 13 or 14 or 17 or 18	76948
20	exp meta analysis/ or exp "systematic review"/	5615
21	(systematic review or meta-analysis).ti,ab,hw.	58362
22	meta-analysis/ or systematic review/ or systematic reviews as topic/ or meta-analysis as topic/ or "meta analysis (topic)"/ or "systematic review (topic)"/ or exp technology assessment, biomedical/ or network meta-analysis/	5615
23	((systematic* adj3 (review* or overview*)) or (methodologic* adj3 (review* or overview*))).ti,ab,hw.	43275
24	((quantitative adj3 (review* or overview* or synthes*)) or (research adj3 (integrati* or overview*))).ti,ab,hw.	10042
25	((integrative adj3 (review* or overview*)) or (collaborative adj3 (review* or overview*)) or (pool* adj3 analy*)).ti,ab,hw.	5461
26	(data synthes* or data extraction* or data abstraction*).ti,ab,hw.	3051
27	(handsearch* or hand search*).ti,ab,hw.	1395
28	(mantel haenszel or peto or der simonian or dersimonian or fixed effect* or latin square*).ti,ab,hw.	5363
29	(met analy* or metanaly* or technology assessment* or HTA or HTAs or technology overview* or technology appraisal*).ti,ab,hw.	936
30	(meta regression* or metaregression*).ti,ab,hw.	2060

31	(meta-analy* or metaanaly* or systematic review* or biomedical technology assessment* or bio-medical technology assessment*).mp,hw.	69387
32	(medline or cochrane or pubmed or medlars or embase or cinahl).ti,ab,hw.	29041
33	(cochrane or (health adj2 technology assessment) or evidence report).jw.	0
34	(comparative adj3 (efficacy or effectiveness)).ti,ab,hw.	2174
35	(outcomes research or relative effectiveness).ti,ab,hw.	3703
36	((indirect or indirect treatment or mixed-treatment or bayesian) adj3 comparison*).ti,ab,hw.	472
37	(meta-analysis or systematic review).md.	51431
38	(multi* adj3 treatment adj3 comparison*).ti,ab,hw.	46
39	(mixed adj3 treatment adj3 (meta-analy* or metaanaly*)).ti,ab,hw.	19
40	umbrella review*.ti,ab,hw.	139
41	(multi* adj2 paramet* adj2 evidence adj2 synthesis).ti,ab,hw.	2
42	(multiparamet* adj2 evidence adj2 synthesis).ti,ab,hw.	6
43	(multi-paramet* adj2 evidence adj2 synthesis).ti,ab,hw.	2
44	or/20-43	113404
45	3 and 19 and 44	233

CDSR

Date Run: 07/10/2021 22:33:34

ID Search Hits
#1 MeSH descriptor: [Dementia] explode all trees 6314
#2 dement* or Alzheim* 29411
#3 #1 OR #2 29568
#4 MeSH descriptor: [Heart Failure] explode all trees 9974
#5 (myocard* or cardia* or heart or ventric*) NEXT (failure or decompensation or insufficien* or dysfunction*) 36145
#6 cardiomyopath* 4633
#7 MeSH descriptor: [Hypertension] explode all trees 19200
#8 hypertens* or "blood pressure" 135375
#9 MeSH descriptor: [Blood Pressure Monitoring, Ambulatory] explode all trees 1517
#10 "24-hour blood pressure monitoring" or "home blood pressure monitoring"328
#11 MeSH descriptor: [Arrhythmias, Cardiac] explode all trees 9989
#12 "atrial fibrillation" or "atrial flutter" or AFIB or arrhythmi* or AF 33281
#13 MeSH descriptor: [Vascular Stiffness] explode all trees 607
#14 "arterial stiffness" or "pulse pressure" or "wave reflections" or "augmentation index" or "pulse wave velocity" or "aortic stiffness" or "central pressure" or "arterial compliance" 5715
#15 MeSH descriptor: [Myocardial Ischemia] explode all trees 29189
#16 ((isch*mic or coronary) and (heart or arter* or disease or syndrome)) or myocard* or angina 95591
#17 MeSH descriptor: [Hyperlipidemias] explode all trees 6571
#18 hyperlipid*mia or dyslipid*mia or hypercholesterol*mia or cholesterol or HDL or LDL 51003
#19 #4 OR #5 OR #6 OR #7 OR #8 OR #9 OR #10 OR #11 OR #12 OR #13 OR #14 OR #15 OR #16 OR #17 OR #18 279224
#20 #3 AND #19 3232

In total 334 reviews found, 45 protocols, 2851 trials, 1 editorial, 1 special collection.

Supplementary Table 2. Data extracted from included meta-analyses

Author and date	Cardiovascular variable	Dementia outcome	No. studies in meta-analysis	Age at baseline (years)	Age stratification	Follow-up (Range)	Total sample size (dementia cases)	Ratio used	Result (95%CI)	p-value	I2 (%)	p-value I2	Notes
**CHD**													
Deckers 2017	CHD	All-cause dementia	7	≥ 45	NR	NR	NR	OR	1.55 (1.20 - 1.84)	0.001	40.6	0.121	
Liang 2021 (1)	CHD	AD	12	NR	NR	NR	NR	RR	0.99 (0.92 - 1.07)	NR	49.8	0.025	
Liang 2021 (4)	CHD	VaD	4	NR	NR	NR	NR	RR	1.34 (1.28 - 1.39)	NR	36.1	0.196	
Liang 2021 (2)	CHD (MI)	AD	7	NR	NR	NR	NR	RR	1.09 (0.90 - 1.33)	NR	41.7	0.113	
Liang 2021 (3)	CHD (AP)	AD	3	NR	NR	NR	NR	RR	0.98 (0.79 - 1.22)	NR	65.4	0.055	
Wolters 2018 (3)	CHD	All-cause dementia	13	62.1 - 81.5	Late-life	2 - 9.3	1,309,483	RR	1.27 (1.08 - 1.50)	NR	80	0.0001	
Wolters 2018 (4)	CHD	All-cause dementia	9	NR	NR	NR	NR	RR	1.26 (1.08 - 1.47)	NR	0	NR	Population-based cohorts only
Wolters 2018 (1)	CHD	AD	8	NR	NR	NR	NR	RR	1.07 (0.90 - 1.28)	NR	31	NR	
Wolters 2018 (2)	CHD	AD	8	NR	NR	NR	NR	RR	1.23 (1.01 - 1.50)	NR	NR	NR	Population-based cohorts only
**HF**													
Li 2020 (2)	HF	All-cause dementia	20	NR	NR	NR	2,406,680	OR/RR	1.28 (1.15 - 1.43)	NR	70	<0.001	
Li 2020 (1)	HF	AD	4	NR	NR	NR	1,950,347	OR/RR	1.38 (0.90 - 2.13)	NR	74.8	0.008	
Wolters 2018 (2)	HF	All-cause dementia	7	69.0 - 81.5	Late-life	4 - 8.6	1,958,702	RR	1.59 (1.19 - 2.13)	NR	58	NR	
Wolters 2018 (3)	HF	All-cause dementia	4	NR	NR	NR	NR	RR	1.80 (1.41 - 2.32)	NR	0	NR	Population-based cohorts only
Wolters 2018 (1)	HF	AD	5	NR	NR	NR	NR	RR	1.44 (0.95 - 2.16)	NR	74	NR	
Xu 2015	HF	AD	3	NR	NR	NR	145,555	OR/RR	1.34 (0.50 - 2.19)	NR	71.3	0.031	
**AF**													
Islam 2019 (2)	AF	All-cause dementia	16	>18	NR	1.8 - 26.6	2,415,35 (200,653)	HR	1.36 (1.23 - 1.51)	<0.001	83.6	NR	
Islam 2019 (1)	AF	AD	6	>18	NR	NR	NR	HR	1.24 (1.03 - 1.49)	0.02	40.5	NR	
Kwok 2011 (1)	AF	All-cause dementia	14	NR	NR	NR	NR	OR	1.98 (1.43 - 2.73)	<0.001	75	<0.001	Overall
Kwok 2011 (2)	AF	All-cause dementia	7	NR	NR	NR	NR	OR	1.64 (1.00 - 2.71)	0.05	87	<0.001	Non-stroke subgroup
Liu 2019	AF	All-cause dementia	11	50.3 - 75.7	NR	1.8 - 26.6	112,876	HR	1.34 (1.24 - 1.44)	NR	0	NR	
Papanastasiou 2021	AF	All-cause dementia	17	NR	NR	NR	NR	HR	1.36 (1.24 - 1.49)	NR	92	<0.001	
Proietti 2020	AF	AD	7	50 - 78	NR	3 - 25 years	56,370	HR	1.30 (1.01 - 1.59)	NR	48.1	0.073	
Santangeli 2012	AF	All-cause dementia	8	61 - 84	Late-life	1.8 - 30.0	77,668	HR	1.42 (1.17 - 1.72)	<0.001	50	NR	
Xu 2015	AF	AD	3	NR	NR	NR	5,240	OR/RR	1.29 (0.97 - 1.60)	NR	60.6	0.079	
Zuin 2021 (2)	AF	All-cause dementia	18	NR	NR	NR	3,559,349 (902,741)	HR	1.40 (1.27 - 1.54)	<0.001	93.5	<0.001	
Zuin 2021 (1)	AF	AD	9	NR	NR	NR	NR	HR	1.30 (1.12–1.51)	<0.001	87.6	<0.001	
**Hypertension**													
Guan 2011	Hypertension (≥160/90mmHg)	AD	9	>40	NR	1 - 32 years	15,292	RR	1.01 (0.87 - 1.18)	NR	37.2	0.12	
Lennon 2019 (1)	Hypertension (SBP>140mmHg)	AD	4	50.4 - 63	Midlife	13 - 22	NR	HR	1.18 (1.02 - 1.35)	0.007	0	NR	
Lennon 2019 (2)	Hypertension (SBP ≥160mmHg)	AD	4	50.4 - 63	Midlife	13.4 - 22	NR	HR	1.25 (1.06 - 1.47)	0.021	0	NR	
Li 2019 (1)	Hypertension (SBP≥160mmHg)	AD	5	35 - 65	Midlife	NR	NR	RR	1.72 (1.25 - 2.37)	NR	39	0.16	
Li 2019 (2)	Hypertension (SBP>140-<160mmHg)	AD	7	35 - 65	Midlife	NR	NR	RR	1.41 (1.23 - 1.62)	NR	10	0.35	
Meng 2014 (1)	Hypertension (≥160/95mmHg)	AD	3	40 - 65	Midlife	14 - 32	NR	OR	1.10 (0.88 - 1.37)	NR	48.6	0.143	
Meng 2014 (2)	Hypertension (SBP ≥160mmHg)	AD	3	40 - 65	Midlife	13.6 - 37	NR	OR	1.77 (0.93 - 3.37)	NR	0	0.48	
Meng 2014 (3)	Hypertension (DBP ≥160mmHg)	AD	3	40 - 65	Midlife	13.6 - 27	NR	OR	2.38 (1.34 - 4.23)	NR	0	0.375	
Meng 2014 (4)	Hypertension (Overall)	AD	5	40 - 65	Midlife	13.6 - 37	NR	OR	1.31 (1.01 - 1.70)	NR	45.7	0.065	
Power 2011 (1)	Hypertension (history and enrollment)	AD	13	58 - 76	NR	NR	NR	RR	0.97 (0.80 - 1.16)	NR	47	NR	
Power 2011 (2)	Hypertension (history and enrollment)	AD	12	>65	Late-life	NR	NR	RR	0.95 (0.78 - 1.14)	NR	NR	NR	
Sharp 2011	Hypertension (history)	VaD	6	NR	NR	3.2 - 10	8,123	OR	1.59 (1.29 - 1.95)	<0.001	37.4	0.16	
Xu 2015 (1)	Hypertension (SBP)	AD	49	NR	NR	NR	2,290,617	OR/RR	0.99 (0.88 - 1.09)	NR	85.5	<0.001	
Xu 2015 (2)	Hypertension (SBP>160mmHg)	AD	6	NR	NR	NR	NR	OR/RR	1.05 (1.01 - 1.08)	NR	0	NR	
Wang 2018 (2)	Hypertension (High SBP)	All-cause dementia	4	<65	Midlife	NR	NR	RR	1.36 (1.06 - 1.75)	NR	43.4	0.151	
Wang 2018 (3)	Hypertension (High DBP)	All-cause dementia	2	<65	Midlife	NR	NR	RR	1.60 (1.02 - 2.53)	NR	23.9	0.252	
Wang 2018 (10)	Hypertension (High SBP)	All-cause dementia	10	≥65	Late-life	NR	NR	RR	1.10 (0.86 - 1.40)	NR	67.5	<0.001	
Wang 2018 (11)	Hypertension (High DBP)	All-cause dementia	7	≥65	Late-life	NR	NR	RR	1.06 (0.68 - 1.65)	NR	67.2	0.006	
Wang 2018 (12)	Hypertension (Not defined)	All-cause dementia	4	≥65	Late-life	NR	NR	RR	0.98 (0.69 - 1.39)	NR	72.1	0.013	
Wang 2018 (13)	Hypertension (High SBP)	All-cause dementia	4	65 - 75	Late-life	NR	NR	RR	0.99 (0.64 - 1.53)	NR	83.6	<0.001	
Wang 2018 (14)	Hypertension (High DBP)	All-cause dementia	3	65 - 75	Late-life	NR	NR	RR	0.73 (0.57 - 0.96)	NR	0	0.877	
Wang 2018 (15)	Hypertension (High SBP)	All-cause dementia	3	75 - 85	Late-life	NR	NR	RR	1.26 (0.98 - 1.63)	NR	0	0.686	
Wang 2018 (16)	Hypertension (High DBP)	All-cause dementia	2	75 - 85	Late-life	NR	NR	RR	0.90 (0.46 - 1.73)	NR	34.4	0.217	
Wang 2018 (1)	Hypertension (High SBP)	AD	2	<65	Midlife	NR	NR	RR	1.50 (0.56 - 4.04)	NR	77.3	0.036	
Wang 2018 (4)	Hypertension (High SBP)	AD	8	≥65	Late-life	NR	NR	RR	1.00 (0.79 - 1.25)	NR	31	0.18	
Wang 2018 (5)	Hypertension (High DBP)	AD	5	≥65	Late-life	NR	NR	RR	0.75 (0.43 - 1.32)	NR	54.5	0.066	
Wang 2018 (6)	Hypertension (High SBP)	AD	3	65 - 75	Late-life	NR	NR	RR	1.01 (0.66 - 1.53)	NR	35	0.215	
Wang 2018 (7)	Hypertension (High DBP)	AD	2	65 - 75	Late-life	NR	NR	RR	0.71 (0.30 - 1.67)	NR	0	0.616	
Wang 2018 (8)	Hypertension (High SBP)	AD	3	75 - 85	Late-life	NR	NR	RR	1.07 (0.63 - 1.82)	NR	66.2	0.052	
Wang 2018 (9)	Hypertension (High DBP)	AD	2	75 - 85	Late-life	NR	NR	RR	0.52 (0.32 - 0.85)	NR	18.7	0.267	
Wang 2018 (17)	Hypertension (High SBP)	VaD	3	≥65	Late-life	NR	NR	RR	1.57 (1.21 - 2.04)	NR	0	0.771	

Wang 2018 (18)	Hypertension (Not defined)	VaD	2	≥65	Late-life	NR	NR	RR	3.69 (1.57 - 8.72)	NR	24.7	0.249	
**Hyperlipidaemia**													
Anstey 2008 (2)	Hyperlipidaemia (TC)	All-cause dementia	6	<60	Midlife	5.6 - 36	4,407	RR	0.97 (0.77 - 1.23)	0.82	NR	NR	Quartiles (Highest vs. lowest)
Anstey 2008 (1)	Hyperlipidaemia (TC)	AD	8	<60	Midlife	4.8 - 29	5,526	RR	0.85 (0.65 - 1.12)	0.24	NR	NR	Quartiles (Highest vs. lowest)
Anstey 2008 (3)	Hyperlipidaemia (TC)	VaD	4	<60	Midlife	4.8 - 29	4,246	RR	1.48 (0.79 - 2.76)	0.22	NR	NR	Quartiles (Highest vs. lowest)
Anstey 2017 (1)	Hyperlipidaemia (TC)	AD	3	40 - 60	Midlife	18 - 32	3,335	RR	2.14 (1.33 - 3.44)	<0.01	NR	NR	Quartiles (Highest vs. lowest)
Anstey 2017 (3)	Hyperlipidaemia (TC)	Dementia	2	40 - 60	Midlife	NR	3,392	RR	1.47 (0.96 - 2.27)	0.08	NR	NR	Quartiles (Highest vs. lowest)
Anstey 2017 (2)	Hyperlipidaemia (TC)	AD/Dementia	4	40 - 60	Midlife	NR	NR	RR	1.82 (1.27 - 2.60)	<0.01	NR	NR	Quartiles (Highest vs. lowest)
Anstey 2017 (4)	Hyperlipidaemia (TC)	AD	4	>60	Late-life	NR	7,308	RR	0.93 (0.69 - 1.26)	0.65	NR	NR	Quartiles (Highest vs. lowest)
Anstey 2017 (6)	Hyperlipidaemia (TC)	VaD	2	>60	Late-life	NR	4,066	RR	0.96 (0.71 - 1.30)	0.79	NR	NR	Quartiles (Highest vs. lowest)
Anstey 2017 (5)	Hyperlipidaemia (TC)	Any dementia	3	>60	Late-life	NR	5,467	RR	1.03 (0.74 - 1.43)	0.88	NR	NR	Quartiles (Highest vs. lowest)
Anstey 2017 (1)	Hyperlipideamia (HDL-C)	AD	3	>60	Late-life	NR	6,062	RR	0.78 (0.54 - 1.13)	0.19	NR	NR	Quartiles (Highest vs. lowest)
Anstey 2017 (2)	Hyperlipideamia (HDL-C)	VaD	2	>60	Late-life	NR	10,558	RR	1.13 (0.60 - 2.14)	0.7	NR	NR	Quartiles (Highest vs. lowest)
Anstey 2017 (3)	Hyperlipideamia (HDL-C)	Any dementia	2	>60	Late-life	NR	5,075	RR	1.06 (0.71 - 1.56)	0.79	NR	NR	Quartiles (Highest vs. lowest)
Anstey 2017 (1)	Hyperlipideamia (TG)	VaD	2	>60	Late-life	NR	10,558	RR	1.66 (0.68 - 4.04)	0.26	NR	NR	Quartiles (Highest vs. lowest)
Li 2019	Hyperlipidaemia (Hypercholesterolemia)	All-cause dementia	4	<65	Midlife	12.8 - 28	NR	RR	1.57 (1.19 - 2.07)	NR	0	0.78	
Meng 2014	Hyperlipidaemia (Hypercholesterolemia)	AD	4	40 - 65	Midlife	21 - 32	NR	OR	1.72 (1.32 - 2.24)	<0.001	8.5	0.351	
Tang 2019 (2)	Hyperlipidaemia (TC)	AD	18	>70	Late-life	NR	NR	SMD	0.05 (-0.09 - 0.20)	0.47	72	<0.001	
Tang 2019 (1)	Hyperlipidaemia (TC Overall)	AD	25	NR	NR	NR	4,920 (2159)	SMD	0.17 (0.01 - 0.32)	0.03	82	<0.001	
Tang 2019 (2)	Hyperlipidaemia (HDL-C)	AD	14	>70	Late-life	NR	NR	SMD	0.03 (-0.26 - 0.19)	0.78	87	<0.001	
Tang 2019 (1)	Hyperlipidaemia (HDL-C Overall)	AD	18	NR	NR	NR	3,968 (1671)	SMD	-0.15 (-0.34 - 0.05)	0.15	87	<0.001	
Tang 2019 (2)	Hyperlipidaemia (LDL-C)	AD	13	>70	Late-life	NR	NR	SMD	0.06 (-0.09 - 0.22)	0.44	70	<0.001	
Tang 2019 (1)	Hyperlipidaemia (LDL-C Overall)	AD	17	NR	NR	NR	3,862 (1627)	SMD	0.18 (-0.02 - 0.38)	0.08	87	<0.001	
Tang 2019 (2)	Hyperlipidaemia (TG)	AD	13	>70	Late-life	NR	NR	SMD	-0.03 (-0.15 - 0.09)	0.64	51	<0.001	
Tang 2019 (1)	Hyperlipidaemia (TG Overall)	AD	17	NR	NR	NR	3,846 (1589)	SMD	-0.00 (-0.12 - 0.12)	1	62	<0.001	
Wu 2019	Hyperlipidaemia (TC)	AD	NR	NR	NR	NR	NR	OR	1.58 (1.10–2.92)	NR	NR	NR	Asian-only population
Wu 2019	Hyperlipidaemia (LDL-C)	AD	NR	NR	NR	NR	NR	OR	1.64 (1.07–2.51)	NR	NR	NR	Asian-only population
Wu 2019	Hyperlipidaemia (HDL-C)	AD	NR	NR	NR	NR	NR	OR	0.81 (0.55–1.19)	NR	NR	NR	Asian-only population
Wu 2019	Hyperlipidaemia (TG)	AD	NR	NR	NR	NR	NR	OR	1.33(0.99–1.79)	NR	NR	NR	Asian-only population
Xu 2015	Hyperlipidaemia (HDL-C)	AD	7	NR	NR	NR	11,991	OR/RR	1.00 (0.86 - 1.14)	NR	0	0.942	
Xu 2015	Hyperlipidaemia (TC)	AD	18	NR	NR	NR	2,246,895	OR/RR	0.96 (0.81 - 1.11)	NR	88	<0.001	
Zhou 2020	Hyperlipidaemia (LDL-C)	AD	26	NR	NR	NR	7,033 (2266)	SMD	0.35 (0.12–0.58)	<0.01	93	<0.001	
**Arterial stiffness**													
Liu 2021 (2)	Arterial stiffness (Aortic PWV categorical)	All-cause dementia	3	>18	NR	1 to 15	NR	OR	2.10 (1.16 - 3.80)	0.014	64.7	0.06	
Liu 2021 (2)	Arterial stiffness (Aortic PWV continuous)	All-cause dementia	5	>18	NR	1 to 15	NR	OR	1.11 (0.98 - 1.25)	0.103	76.6	0.002	

Abbreviations:

AD	Alzheimer's disease
AF	Atrial fibrillation
AP	Angina pectoris
CHD	Coronary heart disease
DBP	Diastolic blood pressure
HDL-C	High-density lipoprotein cholesterol
HF	Heart failure
HR	Hazard ratio
LDL-C	Low-density lipoprotein cholesterol
MI	Myocardial infarction
NR	Not reported
OR	Odds ratio
PWV	Pulse wave velocity
RR	Risk ratio
SBP	Systolic blood pressure
SMD	Standardized mean difference
TC	Total cholesterol
TG	Triglycerides
VaD	Vascular dementia

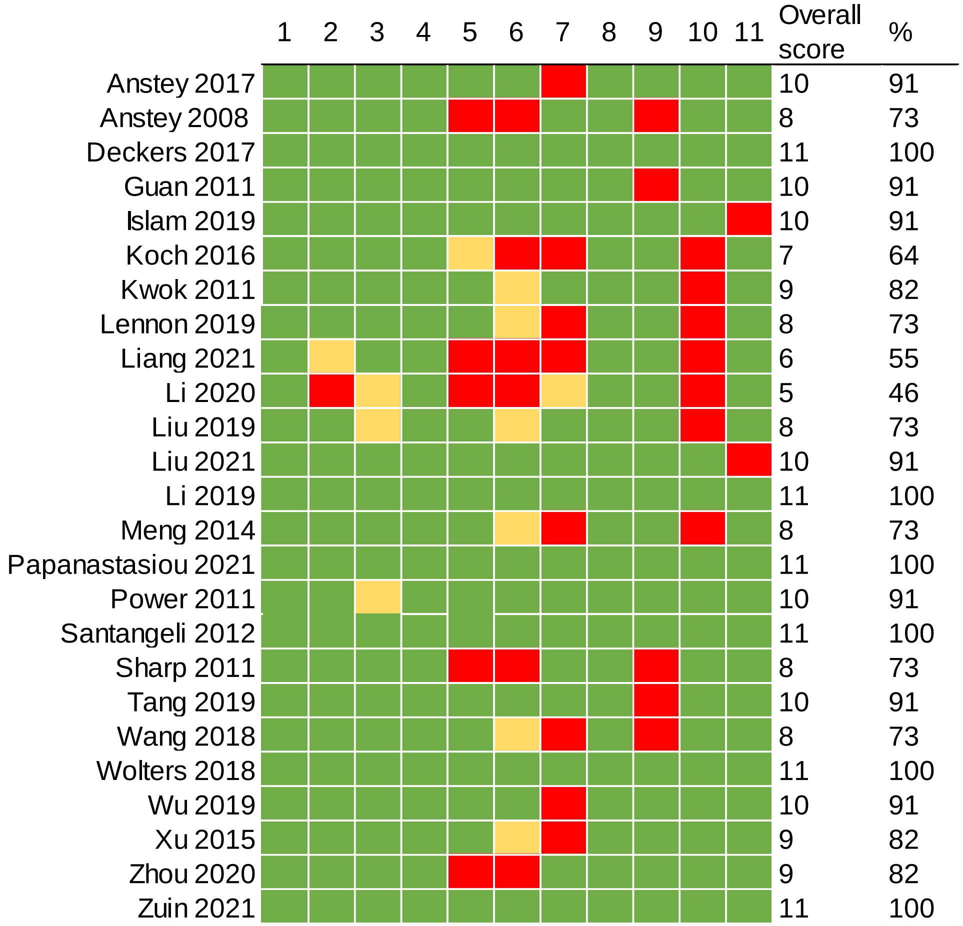

Supplementary Figure 1. Heat map of JBI Critical Appraisal tool for Systematic Reviews. Green represents ‘Yes’, yellow as ‘Unclear’, and red as ‘No’. ‘Yes’ responses are given a score of ‘1’, whilst ‘Unclear’ or ‘No’ given a score of ‘0’. Systematic reviews without a meta-analysis are marked with an asterisk. Q1 Is the review question clearly and explicitly stated, Q2 Were the inclusion criteria appropriate for the review question, Q3 Was the search strategy appropriate, Q4 Were the sources and resources used to search for studies adequate, Q5 Were the criteria for appraising studies appropriate, Q6 Was critical appraisal conducted by two or more reviewers independently, Q7 Were there methods to minimize errors in data extraction, Q8 Were the methods used to combine studies appropriate, Q9 Was the likelihood of publication bias assessed, Q10 Were recommendations for policy and/or practice supported by the reported data, Q11 Were the specific directives for new research appropriate.
